# Downregulation of LOX promotes castration-resistant prostate cancer progression via IGFBP3

**DOI:** 10.7150/jca.61131

**Published:** 2021-10-28

**Authors:** Xuanrong Chen, Yi Shao, Wanqing Wei, Haishan Shen, Yang Li, Yutong Chen, Qianwang Ma, Hanlin Li, Zhao Yang, Yuanjie Niu, Zhiqun Shang

**Affiliations:** 1 Department of Urology, Tianjin Institute of Urology, The second hospital of Tianjin Medical University, Tianjin, 300211, China.; 2 Department of Pediatric Surgery, Huai'an Maternal and Children Health Hospital, Huai'an 223002, China.; 3 Urology Development, Chu Hsien-I Memorial Hospital, Tianjin Medical University, Tianjin, 300211, China.

**Keywords:** lysyl oxidase, IGFBP3, castration resistant prostate cancer, progression

## Abstract

The role of lysyl oxidase (LOX) in prostate cancer remains controversial. Studies have shown that LOX may inhibit the progression of prostate cancer (PCa), whereas other studies demonstrate that LOX may act as a tumor activator in PCa. Here, we report that low LOX expression contributes to CRPC progression through upregulation of IGFBP3. We showed that LOX expression decreased in the more advanced and aggressive castration-resistant prostate cancer (CRPC), compared to castration-sensitive prostate cancer (CSPC). We demonstrated that LOX was negatively correlated with IGFBP3 and may directly bind to the promoter of IGFBP3 and thus decrease the expression of IGFBP3. Inhibition of IGFBP3 by siRNA suppressed the growth and migration of CRPC cells, suggesting a critical role for IGFBP3 in CRPC. The preclinical study in a mouse model suggested that introducing back LOX inhibited the progression of CRPC. In summary, we identified a new function of LOX in PCa and discovered that LOX downregulation contributed to progression via IGFBP3, and that the restoration of LOX may be a promising therapeutic strategy for PCa.

## Introduction

Prostate cancer (PCa) is one of the most common male cancers in the world and the deadliest cancer in the western male population 1. Early-stage prostate cancer can be diagnosed using magnetic resonance imaging and prostate-specific antigen (PSA) screening 2. Patients are usually treated with surgery and radiation therapy and are sensitive to androgen-deprivation therapy (ADT). Unfortunately, these patients eventually developed resistance to ADT treatment, and the development of castration-resistant PCa (CRPC) is inevitable 3.

Lysyl oxidase (LOX) is a copper-dependent amine oxidase and plays a key enzymatic step in the crosslinking of collagen and elastin 4, 5. The C-terminal region of LOX is comprised of a highly conserved, 205-amino acid structural domain that is essential for bioactivity, including four conserved histidine residues coordinating copper binding and a cofactor lysine tyrosine quinone formed by conserved lysine and tyrosine residues. The N-terminal region may determine cellular localization and/or mediate protein-protein interactions 6. Evidence showed that LOX may promote or suppress tumorigenesis, depending on cell type, location, and transformation status 7. LOX plays important roles in cancers such as breast cancer (BCa), liver cancer and gastric cancer 8. Saatci et al. found that LOX was associated with shorter survival in chemotherapy-treated triple-negative BCa patients by promoting the HIF-1α/LOX/ITGA5/FN1 axis 9. Li et al. revealed that LOX could promote liver metastasis in gastric cancer by facilitating the reciprocal interaction between cancer cells and cancer-associated fibroblasts 10. However, the role of LOX in PCa remains unsettled and may have both tumor-suppressing and tumor-promoting effects. Some studies have implicated that LOX was highly expressed and acted as a facilitator in the progression of PCa 11, 12. Other studies demonstrated that LOX acts as a tumor suppressor in PCa 13, 14. These studies primarily concentrate on the level of LOX expression in primary tumors with different Gleason scores, while not shedding light on the exact expression pattern of LOX during the ADT, when tumors developed androgen resistance. In this study, we chose to investigate the role of LOX in CRPC from a more clinically relevant angle.

Insulin-like-growth factor binding protein-3 (IGFBP3) is an N-linked glycosylated, phosphorylated protein, which has been recognized to regulate cancer progression and metastasis 15. Cao et al. revealed that IGFBP3 may activate transcriptional regulation factors in epithelial-mesenchymal transition (EMT), including ZEB1, ZEB2, and Snail and that IGFBP3 loss could suppress the progression of TGF-β1-mediated EMT 16. Hensley et al. found that IGFBP3 was highly expressed in CRPC compared to CSPC and may have predictive value in therapeutically resistant PCa 17. In line with these findings, we found that the expression of IGFBP3 was upregulated in CRPC, correlated to tumor recurrence and poor patient survival. Based on these studies, we hypothesize that LOX deficiency regulates high IGFBP3 expression in CRPC to support progression.

In this study, we show that LOX is downregulated in the CRPC due to its promoter hypermethylation and characterize the role of LOX in PCa cells, and that LOX downregulation contributes to progression via IGFBP3, and that the restoration of LOX may be a promising therapeutic strategy for PCa.

## Materials and Methods

### Patient information and Immunohistochemistry (IHC) assay

All tissue samples were obtained from the Second Hospital of Tianjin Medical University, with the informed consent of all patients. The samples consisted of CSPC patients (n=31) and CRPC patients (n=18) and the detailed information was listed in Supplementary Table 1. All patient samples were collected from men who underwent radical prostatectomy (RP) or transurethral resection of the prostate (TURP), but not in any metastasis regions. The Cambridge dataset comprised fresh frozen samples from 125 primary PCa patients from RP with matched benign tissues, 19 CRPC patients from channel transurethral resection of the prostate (chTURP) 18. Tissue specimens of prostate tumors were formalin-fixed and paraffin-embedded. Serial sections (4 μm) were prepared on charged glass slides. After deparaffinization and rehydration, nonspecific bindings were blocked with antigen-repair solution. Then, sections were incubated with primary antibodies at 4 °C overnight and exposed to second antibodies at 37 °C for 1 hour. Standard DAB staining was carried out to detect IHC targets. Images were captured via a light microscopy.

### Cell culture

The PCa cell line (LNCaP) was acquired from the American Type Culture Collection (ATCC, Manassas, VA, USA). PC3-luc cells were generated by lentivirus-mediated ectopic expression of luciferase protein in PC3 cells. LNCaP-AI cells were described previously 19. Briefly, LNCaP-AI cells were generated from LNCaP cells after long-term castration culture, as a castration-resistant PCa cell line. LNCaP and PC3-luc cells were cultured in RPMI-1640 medium with 10% fetal bovine serum (BI, Cromwell, CT, USA) and 1% penicillin-streptomycin solution. LNCaP-AI was cultured in RPMI-1640 medium with 10% charcoal-stripped fetal bovine serum (BI, Cromwell, CT, USA) and 1% penicillin streptomycin solution. All cells were incubated under the condition of 37 °C with 5% CO_2_.

### Western blot

Cells were washed with cold PBS and lysed with RIPA buffer. Equal amount of protein samples was separated by 10% SDS/PAGE gel and transferred using a PVDF membrane. The membrane was incubated in 5% fat-free milk for 1 hour at room temperature, then incubated with specific primary antibodies overnight at 4 °C. Then membranes were incubated with secondary antibody at room temperature for 1 hour on the next day. After TBST washing, the membrane was prepared for exposure and enhanced chemiluminescence (ECL) reagent (Thermo Scientific, USA) was used to visualize bands. The primary antibodies used as follow: LOX (Sigma, Catalog: 020M1891), IGFBP3 (Proteintech, Catalog: 1758-1-AP), GAPDH (Abways, Catalog: AB0037).

### Methylation specific PCR (MSP)

Genomic DNA of PCa cells (LNCaP and LNCaP-AI) and tissues were extracted using TIANamp Genomic DNA Kits (TIANGEN, Shanghai, China) according to the manufacturer's instruction. The bisulfite modification of genomic DNA was performed using the EpiTect Bisulfite Kits (cat. 59104, Qiagen). For MSP, primers were selected, and performed with general PCR conditions as described before 20.

### RNA interference

Cells were incubated in 6-well plates. Generally, to achieve high transfection efficiency, the cell density was restricted to 40-50%. The RNA oligos were directly transfected using the GP-siRNA-Mate plus transfection reagent (GenePharma) followed by the manufacturer**'**s instructions. The efficiency was determined by mRNA and protein analysis after 2-3 days. The siRNA sequences were listed in Supplementary Table 2.

### RNA analysis and quantitative real-time PCR (Q-PCR)

RNA was isolated using Trizol reagent (Invitrogen) according to the manufacturer's instructions. The cDNAs were obtained by using a reverse transcription kit (Roche). The resulting cDNA was analyzed by PCR using Applied Biosystems 7900 Real-Time PCR System (Thermo Scientific) and SYBR Green PCR Master Mix (Roche) according to the manufacturers' instructions. GAPDH was used as an internal control. The primer sequences were listed in Supplementary Table 2.

### RNA-seq

RNA high throughput sequencing was performed by GENEWIZ Biotech (Suzhou, China). Briefly, total RNA of each sample was extracted using RNeasy Mini Kit (Qiagen). Total RNA of each sample was quantified and qualified by Agilent 2100 Bioanalyzer (Agilent Technologies, USA), NanoDrop (Thermo Fisher Scientific Inc.) and 1% agrose gel. 1 μg total RNA with RIN value above 6.5 was used for following library preparation. The poly(A) mRNA isolation was performed using Poly(A) mRNA Magnetic Isolation Module or rRNA removal Kit. Next generation sequencing library preparations were constructed according to the manufacturer's protocol. Library sequencing was performed on an illumina Novaseq 6000 instrument with 150 bp paired end reads. The DESeq2 (V1.6.3) Bioconductor package was used to perform normalization, then differentially expressed mRNAs were identified by p-value and fold change. The data have been deposited into the China National GeneBank DataBase (CNGBdb) with accession number CNP0001596.

### Chromatin immunoprecipitation (ChIP)

ChIP assay was performed using One-Day chromatin immunoprecipitation Kits according to the manufacturer's instructions (Millipore, Catalog #17-10085). Briefly, cells were cross-linked, lysed and cross-linked protein/DNA complexes were sheared by sonication, and then immunoprecipitated with Anti-Flag (CST, Catalog: 14793) antibody. Then, protein/DNA complexes were eluted and heated to reverse the cross-linking. DNA samples were purified using Spin Columns and analyzed with PCR. The PCR primer sequences were listed in Supplementary Table 2.

### MTT and migration assays

Cells were cultured in 96-well plates. MTT solution (5 mg/ml) was prepared in advance and stored at 4 degrees refrigerator. The cells were incubated in the condition of 37 °C with 5% CO_2_ and harvested for 1-6 days. The optical density OD value of each well was determined by a microplate reader reads at 490 nm wavelength. The migration assay was performed in 24-well culture plates using Transwell chambers (Corning). Cells were cultured in chambers with RPMI-1640 medium and RPMI-1640 medium with 20% fetal bovine serum was placed in each well under the chambers. After 24-48 hours, Transwell chambers were collected to observe the migrated number of cells under a microscope. Each experiment was performed in triplicate and repeated at least three times.

### Animal study

7-week-old nude mice were purchased from Beijing HFK Bioscience Co. Ltd. (Beijing, China). The animal study was approved by the Tianjin Institute of Urology, Tianjin, China. Ten 8-week-old mice were divided into two groups: PC3-luc/rhLOX group (n=5) and PC3-luc/control group (n=5). 5×10^6^ PC3-luc cells were injected by tail vein to achieve tumor metastasis in all groups at Week 10. In the tail vein experiment operation, one nude mouse in the experimental group was deceased, leading to the final number of the experimental group to four. And PC3-luc/rhLOX group (n=4) were treated with rhLOX protein (25 ug/kg; ORIGENE**,** Catalog: TP313323). The PC3-luc/control group (n=4) were treated with vehicle according to a similar dosing schedule. The body weight of each mouse was monitored twice a week. Vehicle and rhLOX protein were administered by peritoneal injection twice a week, for 3 weeks (Week 8-10). At the end of the treatments, the tumor metastasis was observed using Imaging System (IVIS).

### Statistical analysis

Statistical significance was determined using either one-way ANOVA, two-way ANOVA with post hoc multiple comparisons test or unpaired two-tailed Student's t-test for most experiments or otherwise mentioned. P-value < 0.05 was considered significant.

## Results

### LOX is poorly expressed in CRPC and is associated with promoter hypermethylation

**Figure 1:**
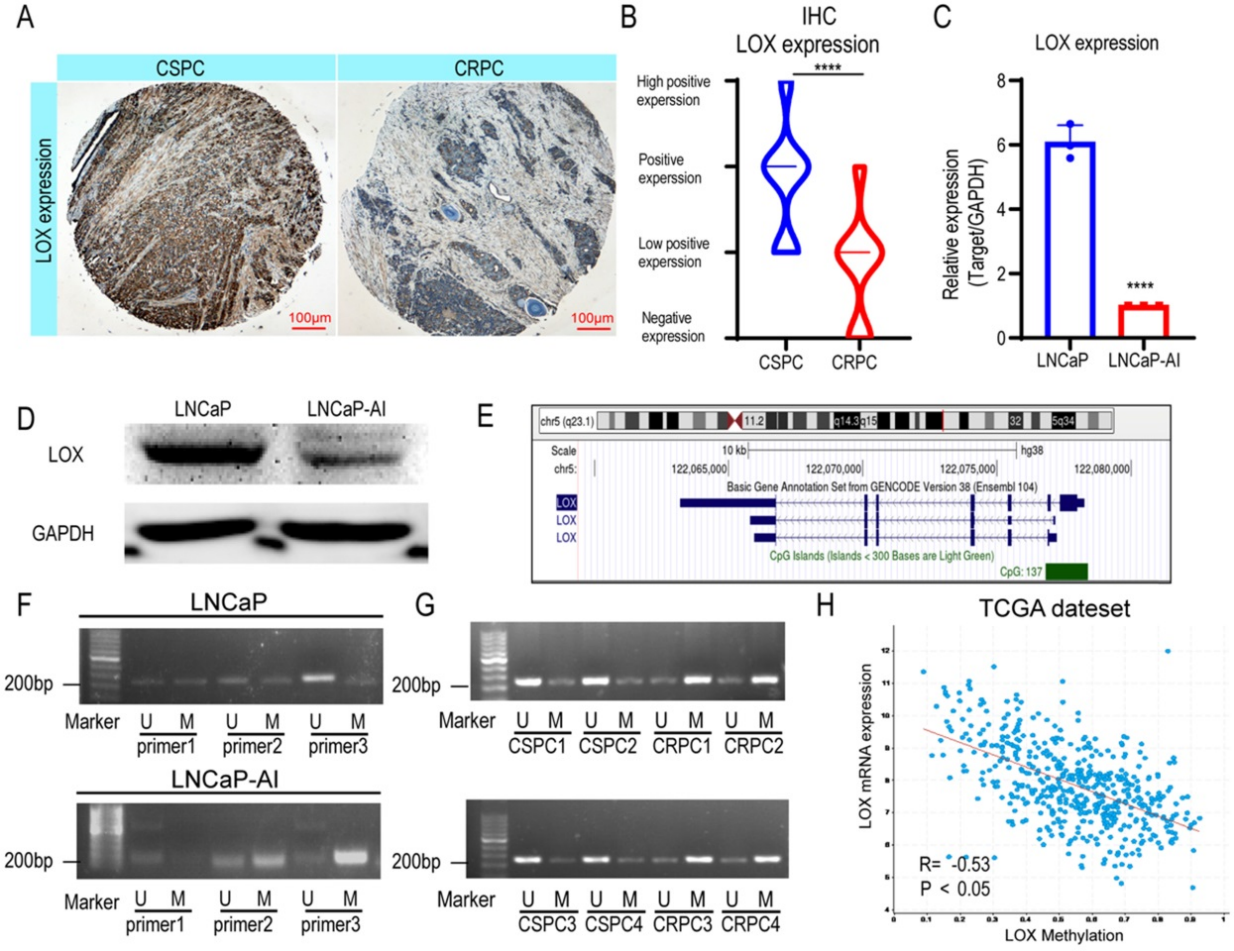
** LOX is poorly expressed in the CRPC due to hypermethylation of its promoter. (A)** IHC assay of LOX expression in both CSPC and CRPC tissues. **(B)** Statistical analysis of LOX expression levels in CSPC and CRPC tissues. **(C)** qRT-PCR detection of LOX expression in LNCaP and LNCaP-AI cell lines, normalized by GAPDH. **(D)** Western blot analysis of LOX protein expression in LNCaP and LNCaP-AI cells. **(E)** The CpG islands of LOX gene. **(F)** MSP analysis of LOX promoter region. **(G)** MSP analysis of LOX promoter region in four CSPC tissues and four CRPC tissues, respectively.** (H)** The association between methylation value of LOX and LOX mRNA expression in the TCGA dataset. U, unmethylated-specific primer; M, methylated-specific primer. For panels **B**, **C**, two-tailed Student's t-test. Error bar indicates the standard deviation (SD). ****, *P* < 0.0001.

Previously, studies revealed that the expression patterns of LOX differed in types and stages of PCa; however, the expression pattern in the CRPC settings remains unclear 11. To address whether LOX expression differs during CRPC progression, we generated immunohistochemistry analyses in our cohort (CSPC patients, n=31; CRPC patients, n=18) (Fig. 1A and Supplementary Table 1). The results showed that LOX was less expressed in the CRPC group when compared with the CSPC group (Fig. 1B). We also performed LOX gene expression profiles on the Cambridge dataset to further evaluate the dysregulation (Supplementary Figure 1A). In the Cambridge dataset, we confirmed that LOX expression was significantly downregulated in CRPC tissues from channel transurethral resection of the prostate compared to primary PCa tissues from radical prostatectomy (*P*<0.05), which consistent with our findings 18. A similar result was also observed in our cell line model. In line with IHC, LOX was down-regulated in LNCaP-AI cells, a CRPC cell line generated from long-term castration culture from parental LNCaP cells, at both RNA and protein levels (Fig. 1C and 1D). Thus, our data suggested that LOX was poorly expressed in the CRPC.

DNA methylation in promoters is well known to correlate with gene expression 21. To examine whether promoter hypermethylation is involved in the low expression pattern of LOX in CPRC, we first investigated the LOX gene loci in the UCSC Genome Browser and found 137 CpG regions in the LOX promoter region of the human genome, indicating the involvement of promoter hypermethylation in LOX expression regulation (Fig. 1E). We then designed specific primers of LOX for methylation-specific PCR (MSP) using MethPrimer to test this hypothesis 22. The results showed that the promoter region of LOX was significantly hypermethylated in LNCaP-AI cells at the site of primer 3, in contrast to LNCaP cells (Fig. 1F). This is also consistent with our findings in patient specimens that were found to be hypermethylated in CRPC tissues in the same region (four CSPC tissues and four CRPC tissues were selected for MSP analysis) (Fig. 1G). Additionally, we also found that the mRNA expression level of LOX was negatively correlated with promoter methylation patterns in the TCGA dataset, further supporting our hypothesis that low expression of LOX in CRPC was correlated with DNA methylation in its promoter regions (Fig. 1H). As the LOX promoter methylation level increases, the LOX mRNA expression level decreases. In summary, we suggested that LOX was downregulated in CRPC owing to its high promoter methylation level.

### LOX inhibits PCa cell migration *in vitro* and *in vivo*

**Figure 2:**
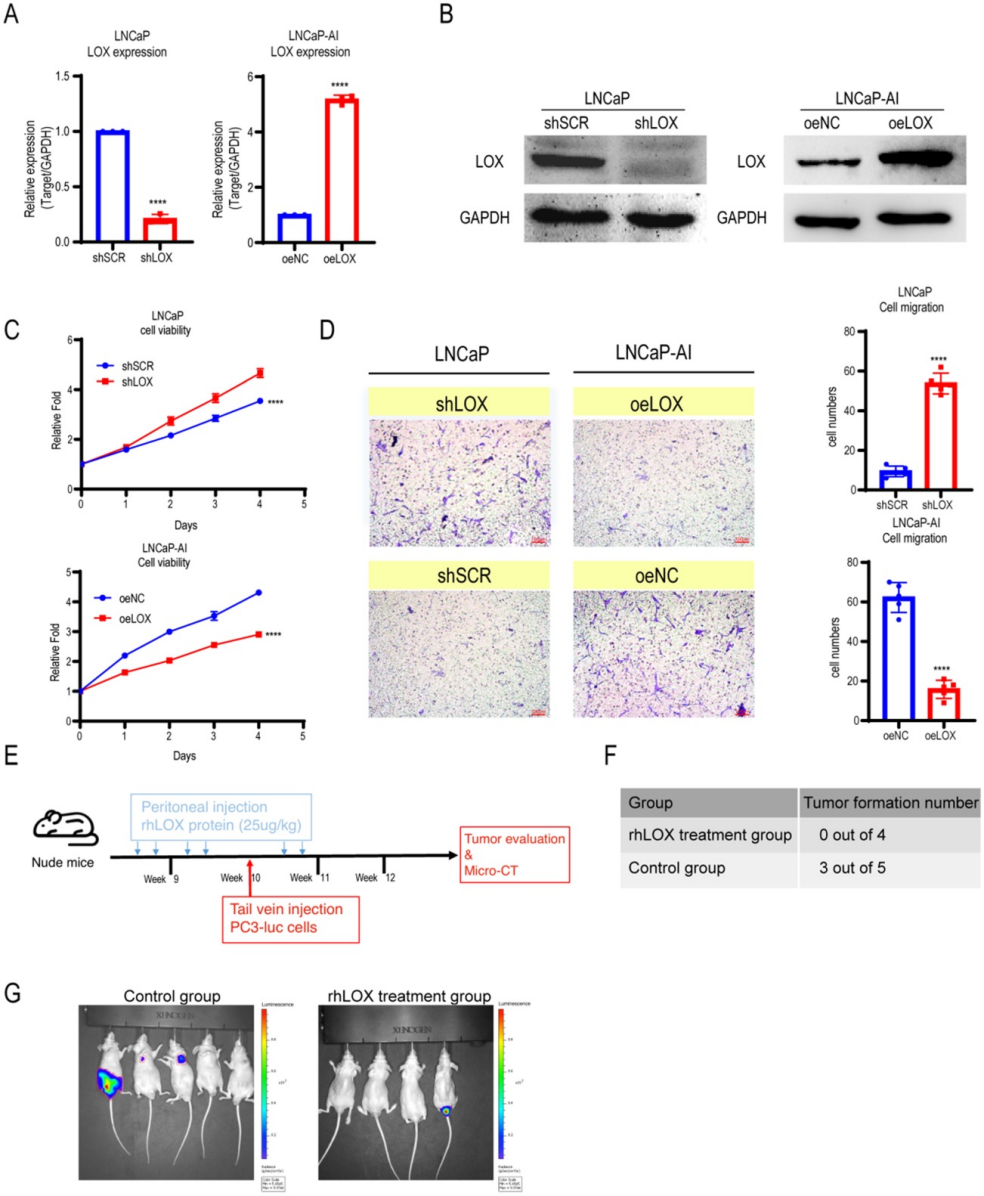
** LOX inhibits the growth and migration of PCa cells. (A)** qRT-PCR detection of LOX expression in LNCaP and LNCaP-AI cells. Left panel: LNCaP cells infected with lentivirus carrying shLOX (LOX-silencing) and control (shSCR). Right panel: LNCaP-AI cells infected with lentivirus carrying oeLOX (LOX-overexpression) and control (oeNC).** (B)** Western blot assay in LNCaP and LNCaP-AI cells. Left panel: LNCaP cells infected with lentivirus carrying shLOX and control. Right panel: LNCaP-AI cells infected with lentivirus carrying oeLOX and control.** (C)** MTT assays. **(D)** Migration assays. The results of the migration assay were quantified with a bar chart. **(E)** Overview of the animal study.** (F)** Tumor formation numbers in the rhLOX treatment group and control group, respectively. **(G)** Bioluminescence measurement images of tumor metastasis in mice. The red dotted line depicts the tumor. For panels **A**, **C**, **D**, two-tailed Student's t-test. Error bar indicates the standard deviation (SD). ****, *P* < 0.0001.

LOX is involved in regulating tumor cell proliferation, supporting metastasis and plays an important role in tumorigenesis and progression 5. To address whether LOX affects PCa progression, we established ectopically re-expressed LOX (oeLOX) and corresponding control (oeNC) stable LNCaP-AI cells, and LOX knockdown (shLOX) and corresponding control (shSCR) stable LNCaP cells (Fig. 2A and 2B). LOX reconstitution drastically inhibited cell proliferation and cell migration in LNCaP-AI cells as shown by the cell growth curve and Transwell assays (Fig. 2C and 2D). A corresponding effect on cell proliferation and migration was observed in LOX knockdown LNCaP cells, suggesting that LOX plays an important role in mediating cell growth and migration, at least in the two examined cell lines. To confirm these observations *in vivo*, we used a xenograft model and added the LOX recombinant protein before and after the tail vein injection of tumor cells to observe the potential effect of LOX on tumors (Fig. 2E). Nude mice (n=4) were injected with rhLOX protein (LOX recombinant protein; 25 ug/kg) by peritoneal injection before and after the tail vein injection of tumor cells within 3 weeks. Another group (n=5) was given the vehicle following a similar dosing schedule as the control group. At week 10, all groups of mice were injected with PC3-luc cells from the tail vein. As observed, the probability of tumor metastasis in the rhLOX treatment group was lower than in the control group, which was consistent with the *in vitro* cell migration results (Fig. 2F and 2G). Moreover, there was also no significant difference in body weight between the rhLOX group and the control group (Supplementary Figure 2). The gross toxic effect of rhLOX treatment on mice was ruled out in the present study. Taken together, we showed that LOX inhibited PCa cell migration both *in vitro* and *in vivo,* and recombinant expression of LOX in PCa may be a treatment strategy.

### LOX deficiency upregulates IGFBP3 expression to promote CRPC cell migration

**Figure 3:**
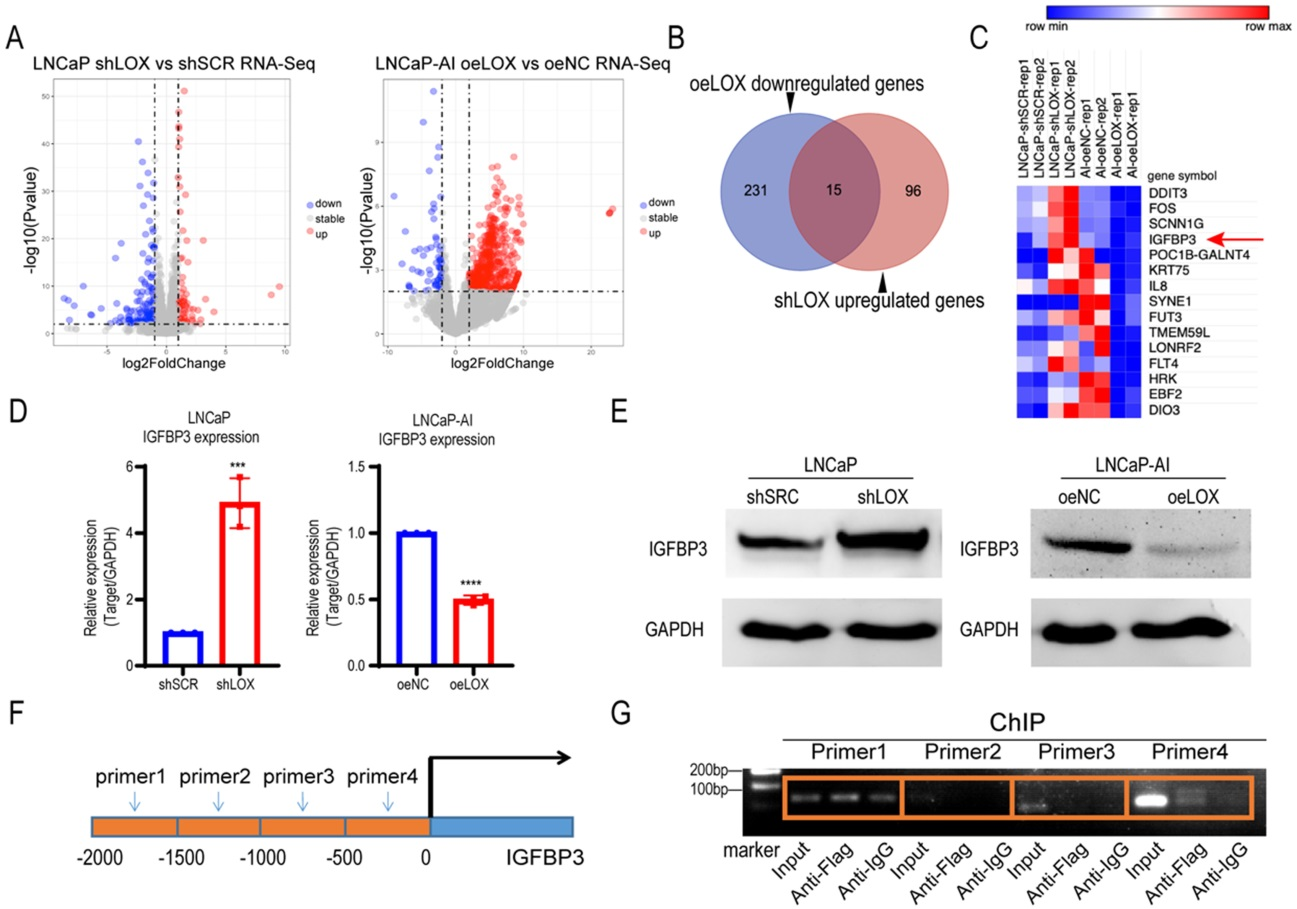
** LOX inhibits the expression of IGFBP3 by binding to its promoter region. (A)** Left panel: Volcano plot of genes significantly upregulated (red) or downregulated (blue) in LOX-silencing LNCaP cells compared to control. Right panel: Volcano plot of genes significantly upregulated (red) or downregulated (blue) in LOX-overexpression LNCaP-AI cells compared to control. Cutoff: Fold change >2.0, *P*<0.05. **(B)** Venn diagram showed oeLOX downregulated genes (n=246) in LNCaP-AI cells interacted with shLOX upregulated genes (n=111) in LNCaP cells. **(C)** Heatmap of genes regulated by LOX in PCa cells. IGFBP3 was marker with a red arrow. **(D)** qRT-PCR detection of IGFBP3 expression in LNCaP and LNCaP-AI cells. Left panel: LNCaP cells infected with lentivirus carrying shLOX (LOX-silencing) and control (shSCR). Right panel: LNCaP-AI cells infected with lentivirus carrying oeLOX (LOX-overexpression) and control (oeNC).** (E)** Western blot detection of IGFBP3 expression. Left panel: LNCaP cells infected with lentivirus carrying shLOX (LOX-silencing) and control (shSCR). Right panel: LNCaP-AI cells infected with lentivirus carrying oeLOX (LOX-overexpression) and control (oeNC).** (F)** Four pairs of primers were designed for the promoter region of IGFBP3. -2000bp to -1500bp of IGFBP3 for primer 1; -1500bp to -1000bp of IGFBP3 for primer 2; -1000bp to -500bp of IGFBP3 for primer 3; -500bp to 0bp of IGFBP3 for primer 4.** (G)** ChIP detection of the interaction between LOX and IGFBP3 promoter region with Flag antibody in oeLOX LNCaP-AI cells. For panel **D**, two-tailed Student's t-test. Error bar indicates the standard deviation (SD). *** *P* < 0.001, and ****, *P* < 0.0001.

Given the biological role of LOX observed above, we sought to explore the potential mechanism of LOX in PCa. To test whether LOX deficiency-induced CRPC progression is caused by alterations in key genes or pathways, we conducted an integrated transcriptome profiling analysis of LOX-silencing LNCaP cells and LOX-overexpressing LNCaP-AI cells using RNA-seq (Fig. 3A). Notably, we found that 15 genes shared the same expression patterns in both LNCaP-AI and LNCaP cells after LOX manipulation (Fig. 3B). We nominated the IGFBP3 gene, which may be targeted by LOX as a downstream gene in PCa (Fig. 3C). We then examined the IGFBP3 expression in our cell lines and found that, consistent with RNA-seq data, the expression of IGFBP3 was dramatically increased in LOX-deficient cells and decreased in LOX-overexpressing cells, coordinated with the inverse of the LOX expression changes (Fig. 3D and 3E). It has been reported that LOX may be recruited to the promoter of target genes and to regulate the expression of the target gene, as confirmed by chromatin immunoprecipitation (ChIP) assays 23. Hence, we wanted to know whether the upregulation of IGFBP3 is mediated by LOX. We designed different paired ChIP primers based on the first 2000 base positions of the IGFBP3 promoter (relative to transcription start site) and found that, in LNCaP-AI cells with ectopic expression of LOX and Flag tag fusion proteins, LOX was directly bonded to the promoter region of IGFBP3 (Fig. 3F and 3G). Taken together, we showed that LOX was recruited to the IGFBP3 promoter to suppress the expression of IGFBP3 in PCa cells.

**Figure 4:**
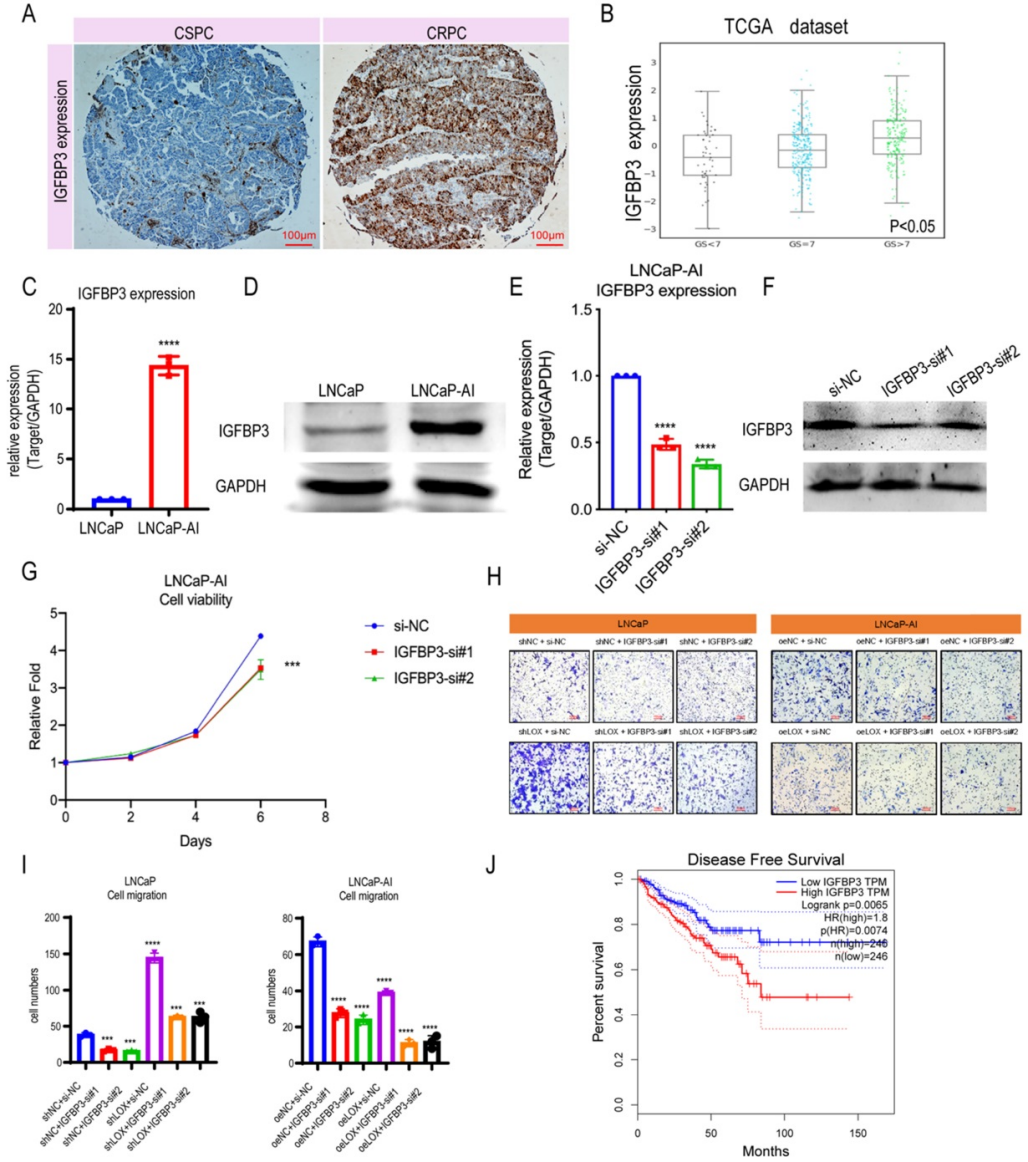
** IGFBP3 predicts a poor prognosis in PCa. (A)** IHC assay of IGFBP3 expression both in CSPC and CRPC tissues. **(B)** IGFBP3 expression in TCGA database. **(C)** qRT-PCR detection of IGFBP3 expression in LNCaP and LNCaP-AI cells**. (D)** Western blot assay detection of IGFBP3 expression in LNCaP and LNCaP-AI cells.** (E)** qRT-PCR detection of IGFBP3 expression in LNCaP-AI cells transfected with IGFBP3-specific siRNAs, respectively.** (F)** Western blot assay of IGFBP3 expression in LNCaP-AI cells transfected with IGFBP3-specific siRNAs, respectively.** (G)** MTT assay in LNCaP-AI cells. **(H)** Rescue assay in LNCaP and LNCaP-AI cells.** (I)** Statistics analysis of rescue assay. **(J)** Disease free survival in IGFBP3 low expression patients and IGFBP3 high expression patients. For panels **B**, **E**, **I**, One-way ANOVA test; for panel **C**, two-tailed Student's t-test; for panel **G**, two-way ANOVA, Sidak's multiple-comparisons test was applied. Error bar indicates the standard deviation (SD). *** *P* < 0.001, and ****, *P* < 0.0001.

Since LOX deficiency upregulates IGFBP3 expression in PCa cells, we next sought to determine whether LOX-regulated IGFBP3 can have a corresponding effect on CRPC progression. We first compared the expression levels of IGFBP3 in PCa and found that the expression of IGFBP3 was significantly upregulated in CRPC patients by the IHC assay, which is consistent with the finding that IGFBP3 may be upregulated in the CRPC owing to the LOX deficiency (Fig. 4A). To further validate, we investigated the IGFBP3 expression pattern in the TCGA database and the Cambridge dataset, and found that IGFBP3 expression increased significantly with tumor progression, and IGFBP3 was significantly upregulated while LOX expression was significantly downregulated in CRPC tissues from channel transurethral resection of the prostate compared to primary PCa tissues from radical prostatectomy (*P*<0.05) (Fig. 4B and Supplementary Figure 1B-C). A similar result was also found in LNCaP-AI cells, where IGFBP3 was dramatically upregulated in LNCaP-AI cells compared to LNCaP cells (Fig. 4C and 4D). We performed IGFBP3 knockdown using two different siRNAs in LNCaP-AI cells and then examined whether IGFBP3 affects CRPC progression (Fig. 4E and 4F). As expected, IGFBP3 knockdown in LNCaP-AI cells significantly disrupted the cell growth and migration (Fig. 4G-I). To verify the function of IGFBP3 in the context of LOX signaling, rescue assays were performed (Fig. 4H and 4I). The results showed that IGFBP3-silencing in LOX-silencing LNCaP cells inhibited cell migration, in contrast to the LOX-silencing group. In addition, IGFBP3-silencing in ectopically re-expressed LOX LNCaP-AI cells also inhibited cell migration. Additionally, patients whose tumors harbored high levels of IGFBP3 had a much shorter disease-free survival time than those whose tumors had low IGFBP3 (log-rank: *p*=0.0065; HR=1.8, *p*=0.0074) (Fig. 4J). Together, these observations indicate that the high expression of IGFBP3 inhibited by LOX due to LOX deficiency in CRPC promotes the progression.

## Discussion

Despite decades of research, the levels of LOX expression and biological function in PCa vary from research to research 5. Several studies have elucidated that LOX expression was significantly reduced in high-grade PCa tissues compared to benign prostate hyperplasia (BPH) using the LOX staining strategy in the tumor cell microenvironment-extracellular matrix (ECM) 14. Additionally, *in situ* hybridization analysis in human prostate tissue and mouse prostate cancer tissue revealed loss of LOX expression during progression, in line with the tumor-suppressor role of LOX 13. Kenyon et al. revealed that LOX may inhibit HRAS-induced tumor formation and reverse HRAS transformation in cancer 24. In nasopharyngeal and bronchogenic carcinoma, results showed that LOX has poor expression, suggesting that LOX may be a suppressor gene for the pathogenesis of the disease 25, 26.

In this study, we demonstrated that LOX was downregulated in the progression to CRPC and verified in our in-house patient samples and *in vitro* LNCaP-derived CRPC cell lines in the laboratory. Of note, all patient samples are from men who have received RP or TURP, but not from any metastatic regions. In contrast, Nilsson et al. found that LOX overexpressed in the bone metastasis regions of mCRPC patients 11. Moreover, patients with high LOX staining activity in non-malignant luminal epithelial prostate tissue had a significantly reduced cancer specific survival compared to the low LOX staining group, but no significant correlation between patient survival and LOX staining activity in tumor epithelium was observed. The differences between Nilsson's and our present study were: 1) Different sample origins. LOX staining was performed mainly in the prostate epithelium, which is consistent with the finding that LOX was largely produced by the prostate epithelium. In our study, LOX expression was significantly downregulated in CRPC tissues from channel transurethral resection of the prostate compared to primary PCa tissues from radical prostatectomy. Nilsson's study investigated the expression of LOX in tumor metastasis areas but did not directly compare the differences in LOX expression between *in situ* CRPC tissues and primary PCa tissues or in the CSPC tissues, which led to the hypothesis that LOX function could differ with context dependent in PCa. 2) Different cell line models. The LNCaP-AI cell line was generated from LNCaP cells after long-term castration culture to mimic clinical androgen-deprived therapy, and our CRPC cell line model showed that LOX expression was downregulated by promoter hypermethylation. Here, we used *in vitro* cell lines and xenograft models to demonstrate that LOX deficiency resulted in CRPC progression, in turn enhancing IGFBP3 expression. Another study demonstrated that IGFBP3 represents a predictive value for treatment resistance in PCa, in accordance with our findings 17. Through our functional analysis, we showed that IGFBP3 may promote CRPC progression and that the upregulation of IGFBP3 may be related to loss of LOX binding to the promoter region in CRPC. Earlier studies showed that administration of the LOX inhibitor, BAPN, started after AT-1 (a rat prostate cancer cell line) tumor cell implantation tended to have no effect, or even increased cell growth 12. While, in our study, by administration of the LOX recombinant protein (rhLOX) before and after the tail vein injection of PC3 tumor cells (a CRPC cell line), we showed that rhLOX treatment may reduce the tumor metastasis probability, and consistent with the *in vitro* cell migration data we presented.

Our study used a model system and human tissue, but there are still limitations to the model system. For example, the xenograft tumor model lacks the tumor environment provided by human prostate stromal cells, making it difficult to observe the LOX activity in the tumor stroma and/or the surrounding tumor-adjacent non-malignant prostate stroma to further address the LOX function in the context dependent tumor model. Further studies are needed to clarify the circumstances under which LOX inhibitors *in vivo* are used to address this possibility. In addition, the human PCa sample used in our study only represents a time point in the disease process. Sampling from the same patient at different stages is ideal for this research, but for many reasons is difficult.

## Supplementary Material

Supplementary figures and tables.
