# Supplementary material for: Downregulation of LOX promotes castration-resistant prostate cancer progression via IGFBP3
Source: J Cancer. 2021 Oct 28;12(24):7349–57. doi: 10.7150/jca.61131 (PMC8734407; doi:10.7150/jca.61131)
Supplement: Supplementary file 1 — Supplementary figures and tables. [file jcav12p7349s1.pdf]

Supplementary information

Downregulation of LOX promotes castration-resistant prostate cancer progression via IGFBP3

Xuanrong Chen[1,*], Yi Shao[1,*], Wanqing Wei[1,2,*], Haishan Shen[3,*], Yang Li[1], Yutong Chen[1], Qianwang Ma[1], Hanlin Li[1], Zhao Yang[1], Yuanjie Niu[1,#], Zhiqun Shang[1,#]

[1]Department of Urology, Tianjin Institute of Urology, The second hospital of Tianjin Medical University, Tianjin, 300211, China.

[2]Department of Pediatric Surgery, Huai'an Maternal and Children Health Hospital, Huai'an 223002, China.

[3]Urology Development, Chu Hsien-I Memorial Hospital, Tianjin Medical University, Tianjin, 300211, China.

[*] These authors contributed equally to this work.

[#]Corresponding author. Zhiqun Shang: zhiqun_shang@tmu.edu.cn or Yuanjie Niu: niuyuanjie9317@163.com

Supplementary Figures 1-2

Supplementary Tables 1-2

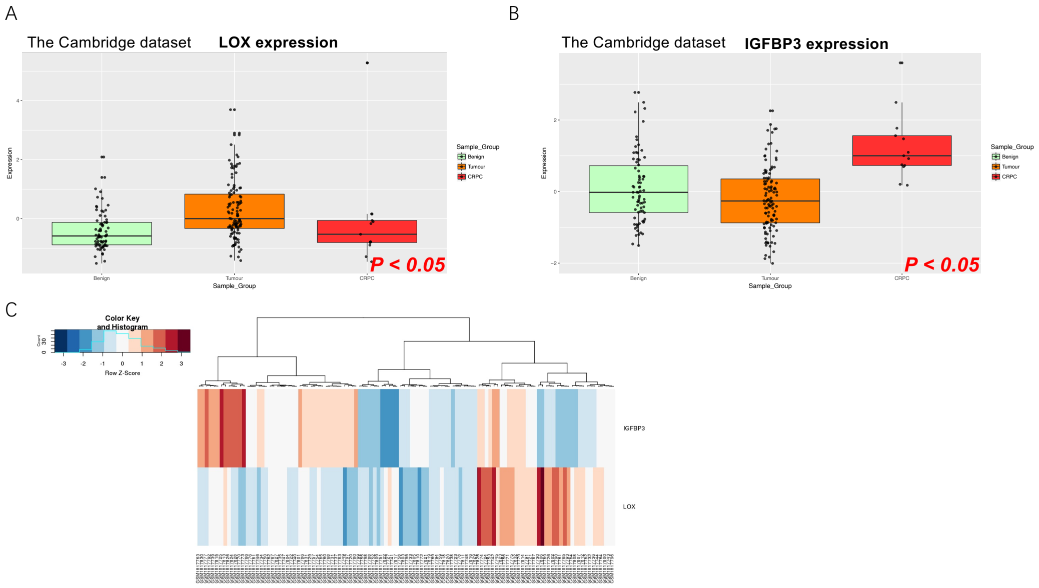

Supplementary Figure 1. Gene expression profiles in the Cambridge dataset.
(A) The expression of LOX gene in the Cambridge dataset by RNA-seq.
(B) The expression of IGFBP3 gene in the Cambridge dataset by RNA-seq.
(C) The heatmap of the selected gene expression profiles. Shades of red and blue represents expression values in z-score form.

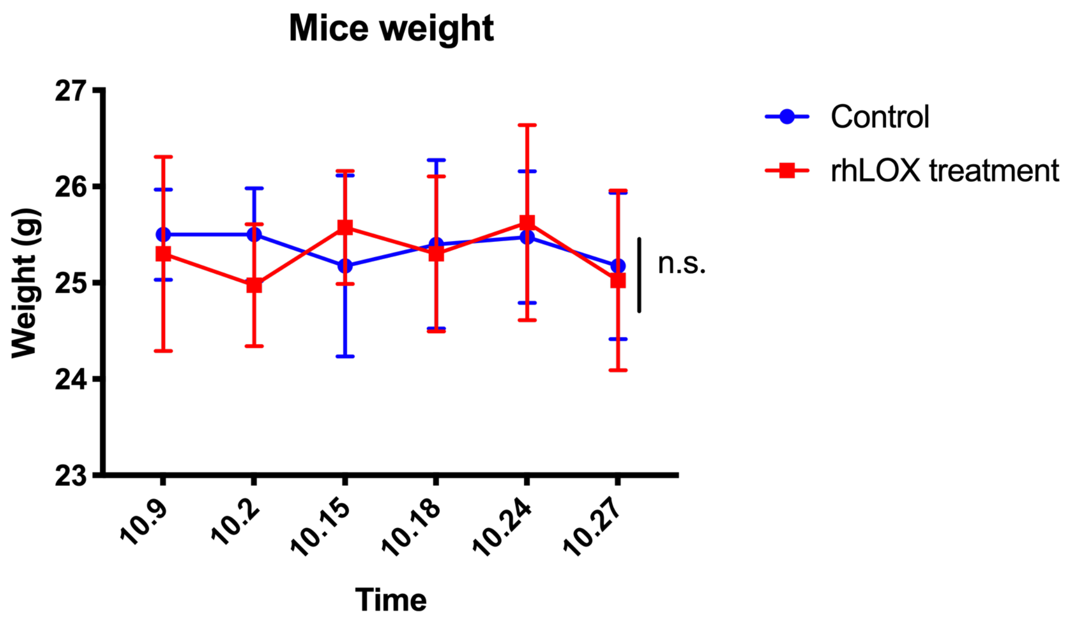

Supplementary Figure 2. Changes in body weight during the study period.

Supplementary Table 1. The main characteristics of the CSPC patients (n=31) and CRPC patients (n=18).

Characteristics	CSPC<br>n	CRPC<br>n
Age		
< 66 years	13	3
≥ 66 years	18	15
Gleason score		
<8	16	3
≥8	15	15
Pathological stage		
< T3A	24	0
≥ T3A	7	18

(CRPC: castration-resistant prostate cancer; CSPC: castration-sensitive prostate cancer; PSA: prostate specific antigen)

Supplementary Table 2. All primer and siRNA sequences.

Gene	Usage	Sequence (5'-3')
LOX sense#1	RNAi	GAAUCUGACUAUACCAACATT
LOX antisense#1	RNAi	UGUUGGUAUAGUCAGAUUCAG
LOX sense#2	RNAi	GCACAGUUGUCAUCAACAUTT
LOX antisense#2	RNAi	AUGUUGAUGACAACUGUGCCA
IGFBP3 sense#1	RNAi	GCAGUGUCGCCCUUCCAAATT
IGFBP3 antisense#1	RNAi	UUUGGAAGGGCGACACUGCTT
IGFBP3 sense#2	RNAi	CAUUCAAAGAUAAUCAUCATT
IGFBP3 antisense#2	RNAi	UGAUGAUUAUCUUUGAAUGGA
GAPDH forward	Q-PCR	GGAGCGAGATCCCTCCAAAAT
GAPDH reverse	Q-PCR	GGCTGTTGTCATACTTCTCATGG
LOX forward	Q-PCR	CGGCGGAGGAAAACTGTCT
LOX reverse	Q-PCR	TCGGCTGGGTAAGAAATCTGA
IGFBP3 forward	Q-PCR	AGAGCACAGATACCCAGAACT
IGFBP3 reverse	Q-PCR	GGTGATTCAGTGTGTCTTCCATT
GAPDH forward	Q-PCR	GGAGCGAGATCCCTCCAAAAT
Primer1-M forward	MSP	AGTAGATTTAATGGGAGAATAACGG
Primer1-M reverse	MSP	GAATCAACAAAATCGAAATACGAA
Primer1-U forward	MSP	AGTAGATTTAATGGGAGAATAATGG
Primer1-U reverse	MSP	CAAATCAACAAAATCAAAATACAAA
Primer2-M forward	MSP	CGAGGAGTTGTTCGTTTTGTAC
Primer2-M reverse	MSP	AAAATTTTAAACTTTCTAACACGTT
Primer2-U forward	MSP	AGGTGAGGAGTTGTTTGTTTTGTAT
Primer2-U reverse	MSP	AAAATTTTAAACTTTCTAACACATT
Primer3-M forward	MSP	AGTAGATTTAATGGGAGAATAACGG
Primer3-M reverse	MSP	GAATCAACAAAATCGAAATACGAA
Primer3-U forward	MSP	GTAGATTTAATGGGAGAATAATGG
Primer3-U reverse	MSP	CAAATCAACAAAATCAAAATACAAA
Primer1-ChIP forward	ChIP	AGACTTCGCCTGCCAACG
Primer1-ChIP reverse	ChIP	CCCTTACCCCTTCCGCTCT
Primer2-ChIP forward	ChIP	ATTTCAACAGCGTTCAGG
Primer2-ChIP reverse	ChIP	TTCGGTGACCAACAGAGG
Primer3-ChIP forward	ChIP	TACCCAAGACAAGAAGAACA
Primer3-ChIP reverse	ChIP	ACACCGCAAGTCTCCAAT
Primer4-ChIP forward	ChIP	GGAACGGATGTAAACCTG
Primer4-ChIP reverse	ChIP	ATAATCGTCGCTTGCTGT

(RNAi: RNA interference; Q-PCR**:** quantitative real-time PCR; MSP: methylation specific PCR; ChIP: chromatin immunoprecipitation)
